# A case–control study examining the relationship between social cognition and post-traumatic stress disorder

**DOI:** 10.1192/bjo.2026.12006

**Published:** 2026-06-04

**Authors:** Chantelle E. Wiseman, Andrew D. Lawrence, Jonathan I. Bisson, Mohamed Bader, Stanley Zammit

**Affiliations:** Department of Population Health Sciences, University of Bristol, UK; School of Philosophy, Psychology & Language Sciences, University of Edinburgh, UK; School of Psychology, Cardiff University, UK; Division of Psychological Medicine and Clinical Neurosciences, Cardiff University, UK; South Caerphilly Community Mental Health Team, Caerphilly, UK; Centre for Academic Mental Health, Division of Population Health Sciences, Bristol Medical School, University of Bristol, UK

**Keywords:** Social cognition, mentalisation, PTSD, epidemiology, trauma and stressor-related disorders

## Abstract

**Background:**

Difficulties with social cognition, particularly with mentalising, have been hypothesised to increase the risk of, and affect recovery from, trauma and post-traumatic stress disorder (PTSD). We lack a comprehensive picture of the nature of social cognitive impairments in people with PTSD.

**Aims:**

To assess whether variation in social cognition is associated with trauma and PTSD.

**Method:**

Ninety-eight participants with PTSD symptoms and 99 controls were recruited from tertiary PTSD services and the Prolific online platform. All participants completed a battery of social cognition tasks covering face emotion recognition, social attribution and mentalisation. They also completed a trauma measure, PTSD screening measure and verbal IQ task. Logistic regression analyses were used to examine the relationship between social cognition and PTSD, and were adjusted for age, gender, autism and verbal IQ.

**Results:**

There was some evidence that hypomentalisation, measured via the Reflective Functioning Questionnaire, was associated with exposure to trauma (adjusted odds ratio 1.80, 95% CI 1.03–3.14, *p* = 0.040) and increased odds of PTSD symptoms (adjusted odds ratio 4.29, 95% CI 2.76–6.66, *p* < 0.001). Higher scores for use of mentalising language on a naturalistic video-based spontaneous mentalising task (Modified-STOMP; potentially reflecting hypermentalising) were also associated with increased odds of PTSD symptoms (adjusted odds ratio 1.65, 95% CI 1.17–2.33, *p* = 0.004) in the main analysis, but not in the sensitivity analysis restricted to the Prolific-only sample.

**Conclusions:**

Our results show an association between mentalising difficulties and PTSD symptoms, indicating mentalising might be a target for future risk prediction.

Post-traumatic stress disorder (PTSD) is a psychiatric condition precipitated by a traumatic event. This event must be severe, involving ‘actual or threatened death, serious injury or sexual violence’.^1^ PTSD is characterised by symptoms of re-experiencing (flashbacks, nightmares, intrusive thoughts), alongside avoidance of reminders of the trauma, negative mood and cognitions, and hyperarousal.^1^ Although trauma exposure is necessary to develop PTSD, most adults are exposed to at least one traumatic event in their lifetime and only a minority develop PTSD.^2^ Difficulties with social cognition have been hypothesised as a risk factor for PTSD.^3^

## Difficulties in social cognition as a risk factor for PTSD

Social cognition is the psychology of understanding our social environment and relationships, as well as our own motivations. Agreement on taxonomy is lacking,^4^ but there do seem to be different constructs within social cognition. Previous work aiming to identify areas for use in clinical studies via expert consensus has identified four domains.^5^ Emotion processing is the ability to identify emotions from facial expressions and vocal prosody, as well as the capability to manage emotions. Social perception refers to having social knowledge of the norms and values of that society and understanding social context. The tendency to attribute the cause of events to the environment, others or the self is attributional style or bias.

Finally, the most complex area of social cognition is mentalisation or theory of mind. This is the ability to understand the mental state of other people and oneself; it is also known as cognitive empathy. Mentalisation requires the ability to integrate the lower-order social cognitive abilities and recognise that the experiences of others may be different from our own. Poorer mentalising can be divided into two broad types: hypomentalising is concrete thinking with difficulty relating to the internal worlds of others, and hypermentalising is excessive but potentially inaccurate mentalising.^6^

More recent work has looked at distinguishing the ability to mentalise from the likelihood of an individual doing so. Mentalising ability is the accuracy of identifying the mental states of others and the self; it can be measured using tasks to determine accuracy.^7^ Mentalising propensity is whether people tend to mentalise or not.^7^ Performance measures and self-reported tendencies are typically only weakly correlated at best.^8^

Problems with social cognition are hypothesised to increase the risk of developing PTSD in several ways.^3,9^ First, individuals with difficulties with social cognition might be more likely to experience interpersonal trauma if they struggle to infer threat during interactions with others. Second, the threshold for what is experienced as a trauma may be lowered: two people experiencing the same event may differ in how upsetting they find this depending on their capacity to understand the social context surrounding this. Third, having poorer mentalisation could result in reduced social support, increasing the likelihood of a traumatic event resulting in PTSD. Fourth, focusing on the well-being of others during the peri-traumatic period at the expense of processing one’s own trauma due to hypermentalising could also increase the risk of PTSD. For further details of our hypotheses please see Wiseman, 2024.^9^

Previous studies, most using cross-sectional data and with small sample sizes, have shown impairments in social cognition in people with PTSD compared with controls. Three review articles on this topic have been published in the past decade, and although there has been heterogeneity in the findings, mentalisation has been consistently shown to be impaired in people with PTSD compared with controls.^10–12^

In this study, we examined whether social cognitive abilities, including mentalising tendencies, are altered in people with PTSD compared with controls. We aimed to build on previously published work by using multiple indices of social cognition to more comprehensively examine this construct, and in a larger sample size than most previous studies.

## Aims

To determine if multiple indices of social cognition are associated with trauma and PTSD.

## Method

### Participants

Participants were recruited from both specialist National Health Service (NHS) PTSD services and the online recruitment platform Prolific.^13^ The case group consisted of people with PTSD (*n* = 98) who were recruited from both clinical (NHS Traumatic Stress Services; *n* = 56) and non-clinical (Prolific online platform; *n* = 42) samples. Controls (*n* = 99) were recruited via Prolific. All participants provided written informed consent for their data to be used in this study. Procedures involving patients were approved by the NHS Ethics Oxford B Research Ethics Committee (approval number IRAS 263222) in 2019, and procedures involving non-clinical participants were approved by Cardiff University School of Psychology Research Ethics Committee in 2021 (approval number EC.21.01.12.6258R2).

### Data collection

Data for the clinical sample were collected between December 2019 and August 2023. All participants completed data collection on the Qualtrics survey platform.^14^ Clinical participants, recruited from two tertiary PTSD treatment centres in the UK, completed this data collection as part of a longitudinal study examining whether pre-therapy social cognition in people with PTSD is associated with recovery.^15^ The data from the baseline assessment in the longitudinal study were used for the current investigation. Inclusion criteria were: 18 years of age or over, a primary diagnosis of PTSD (as recorded in the clinical notes following clinical assessment) and English fluency. The first six clinical sample participants completed data collection using the Qualtrics survey platform, with face-to-face instruction from a member of the research team. The remaining participants completed data collection and written consent remotely because of the COVID-19 pandemic. Participants were remunerated with a £20 gift voucher on completion of follow-up of the longitudinal study.

**Fig. 1:**
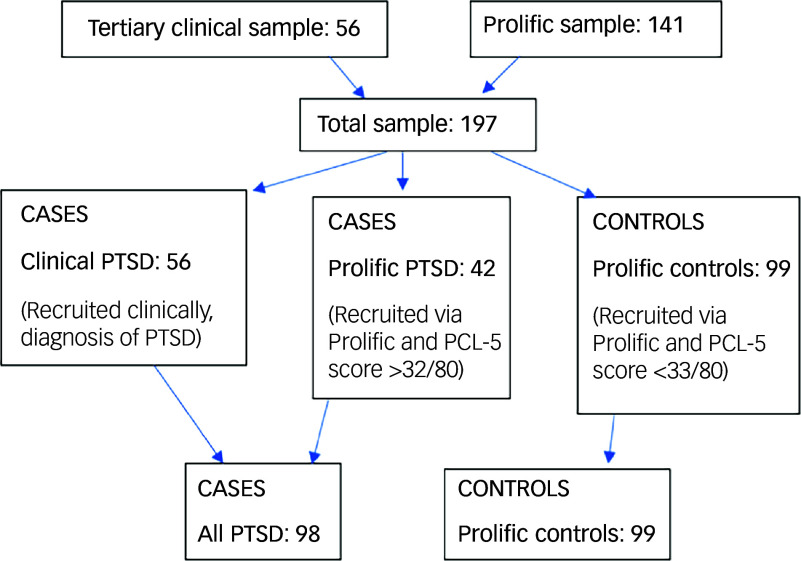
Flowchart of study participants. PCL-5, PTSD Checklist for DSM-5; PTSD, post-traumatic stress disorder.

All Prolific sample participants completed data collection remotely. The study was advertised on Prolific^13^ to participants who fulfilled the inclusion criteria: 18 years of age or over, fluency in English, resident in the UK, with access to an electronic device with a stable internet connection and with sufficient concentration for a 90 min study. Participants completed the tasks and questionnaires on the Qualtrics survey platform, and all passed at least two of three attention checks embedded into the survey. Prolific sample participants were remunerated £10. Of the 141 Prolific sample participants, 42 screened positive for PTSD using the PTSD Checklist for DSM-5 (PCL-5) (described below) and were therefore included as part of the PTSD case group, along with the 56 cases recruited from the clinical sample (all PTSD, *n* = 98). The Prolific PTSD sample did not receive a clinical assessment for PTSD. They were categorised into this group using the screening questionnaire PCL-5. The remaining 99 Prolific participants were the control group (Prolific controls). See Fig. 1 for details.

The authors assert that all procedures contributing to this work comply with the ethical standards of the relevant national and institutional committees on human experimentation and with the Helsinki Declaration of 1975, as revised in 2013.

### Measures

All measures were completed by participants on Qualtrics.^14^ Please see our protocol paper of the longitudinal study for more detail.^15^

### Exposures

The Social Cognition in PTSD battery consists of five social cognition measures, described briefly below.

#### Social Shapes Test

The Social Shapes Test (SST) measures mental state attribution. Participants view 23 short video clips about animated coloured shapes interacting and must pick from one of four options when asked a question about the scene (e.g. ‘The other shapes don’t like blue triangle’).^16^ Total score was the number of correct trials.

#### Emotion odd-one-out ‘Oddity’ task

This task, referred to as the Oddity task, measures face emotion recognition.^15^ For each of the 36 trials, three images of faces are shown, of which two show the same emotion and one shows a different emotion. Participants must pick the odd one out. Total score was the number of correct trials.

#### Reading the Mind in the Eyes Task

The Reading the Mind in the Eyes Task (RMET) measures emotion recognition and mentalising.^17,18^ Participants are given 36 trials of pictures of eyes with four possible mental states for each. They must select the appropriate mental state.^19^ Total score was the number of correct trials.

#### Spontaneous Theory of Mind Task

The Spontaneous Theory of Mind Task (STOMP) is is measure of spontaneous mental state attribution. A 90 s video clip from a romantic comedy is shown with no sound and participants are then asked to ‘Please describe this scene’ in around seven to ten lines of text.^20^

We used the Linguistic Inquiry and Word Count (LIWC) software to analyse the STOMP task.^21^ Words used can give information about the psychological states of individuals.^21^ The LIWC is an extensively validated text analysis software that can provide scores on different aspects of text for various linguistic dimensions, psychological processes and types of words. Although the LIWC can be programmed to count any words, we focused our analysis on linguistic content indicative of social reasoning. Specifically, we used the proportion of ‘Insight’ words of the written texts for the STOMP task as an index of mentalising. Insight is categorised by the LIWC as a psychological process that measures words relating to ‘know, how, think, feel’, and higher proportions are indicative of greater density of this language in the text. Prior research has shown that ‘Insight’ words are powerful markers of efforts to cognitively appraise and make sense of experiences and events, including traumatic events and complex social narratives.^22–24^ Because this is a different scoring approach to that used by the authors of the STOMP task, we refer to this as the Modified-STOMP.

#### Eight-Item Reflective Functioning Questionnaire

The Eight-Item Reflective Functioning Questionnaire (RFQ-8) is is a self-report measure of dispositional difficulties with mentalising. This questionnaire was designed to measure both the propensity for hypomentalisation (concrete thinking) and the propensity for hypermentalisation (confidence in and over-interpretation of neutral mental states).^6^ Participants respond on a Likert scale to eight statements. Six of the statements are scored to produce the hypomentalisation measure (RFQ-U) and six to produce the hypermentalisation measure (RFQ-C), using a scoring process described by the authors.^6^ We reverse coded the RFQ-C so for both measures a higher score was indicative of greater difficulties mentalising. Because of the strong collinearity between the RFQ-U and RFQ-C (see below), we used only the RFQ-U for our regression analyses.

### Outcomes

#### Life Events Checklist for DSM-V

The Life Events Checklist for DSM-V (LEC-5) questionnaire records lifetime exposure to 17 different traumas, including serious accidents, physical assaults and life-threatening illness or injury.^25^ We used a modified version that includes two additional items on childhood abuse (sexual and physical). Participants record if they have experienced the trauma, witnessed it, learnt about someone close to them experiencing it or were exposed to it as part of their job. We computed a binary score of trauma being ever experienced (yes/no).

#### PCL-5

The PCL-5 checklist measures the 20 symptom criteria of DSM-5 defined PTSD over the past month^26^ on a Likert scale from 0 to 4. Total scores range from 0 to 80, and we used a score of 33 or greater as indicative of a positive screen for PTSD.^26^

### Confounders

There are gender differences in emotion recognition abilities and empathy,^27,28^ and PTSD prevalence is higher in women.^29^ Social cognition varies across the lifespan as social roles and demands change, and older adults can have difficulties in emotion recognition and complex social cognitive abilities.^30,31^ PTSD incidence also varies by age with greatest prevalence in the middle years of life.^32,33^ Verbal ability and education are positively correlated with social cognitive performance,^16,17^ and lower IQ is associated with PTSD.^34^ Impaired social cognition is a hallmark of autism,^35^ and people with autism have a higher incidence of PTSD.^36^ We therefore adjusted for verbal IQ, age, gender and autism as confounders in our regression analyses.

#### Spot-the-Word task

This is a brief verbal IQ task that consists of 60 dyads of a real word and a fake word. Participants must identify the real word in the pair.^37^ It has good concurrent validity with other measures of premorbid intelligence.

### Other confounders

Data on age, gender and other psychiatric diagnoses, including autism, were self-reported by the Prolific sample and obtained from the clinical notes for the clinical sample.

### Data analysis

Stata version 17 for Windows (StataCorp, College Station, Texas, USA; https://www.stata.com/) was used for statistical analyses.

Differences in gender, age, performance on the social cognition measures and autism diagnoses were compared between the all PTSD case group and Prolific control group. Chi-squared tests were used to compare categorical measures. The Mann–Whitney *U*-test was used to compare the medians of non-parametric data. Spearman’s rank correlation was used to investigate the relations between the social cognition variables, as scores were mostly non-normally distributed with a left skew. Three-group comparisons were also made between the clinical PTSD, prolific PTSD and Prolific control groups, using the chi-squared and Kruskal–Wallis tests.

Logistic regression analyses were used to determine whether the social cognition indices were associated with odds of trauma or PTSD (SST, Oddity, RMET, Modified-STOMP and RFQ-U, but not the RFQ-C because of collinearity issues, see below). Standardised social cognition measures were used for the regression analyses. Multivariable logistic regression was used to adjust for confounders. We present the odds ratio, 95% confidence interval and *p*-values for these analyses. A sensitivity analysis was completed repeating the regression analyses for the outcome of PTSD restricted to the Prolific sample only.

Missing data were minimal, at <5%.

## Results

### Demographics

There were 197 participants included in this study. The age range was from 18 to 61 years, with a mean age of 35 years. Fifty-five (27.9%) participants were men; 176 (89.3%) participants had experienced a trauma. See Table 1 for further details.

**Table 1:** Comparison of the clinical and Prolific sample

Characteristic	Whole sample (s.d.)	Clinical sample (s.d.)	Prolific sample (s.d.)
Age, years, mean	35.30 (11.76)	38.29 (11.50)	34.11 (11.70)
PCL-5 score	34.34 (24.10)	61.52 (9.78)	23.54 (19.04)
Spot-the-Word score	49.55 (5.75)	48.77 (5.56)	49.87 (5.81)

	Whole sample (%)	Clinical sample (%)	Prolific sample (%)
Male	55 (27.9)	12 (21.4)	43 (30.5)
Trauma exposure	176 (89.3)	56 (100)	120 (85.1)

PCL-5, PTSD Checklist for DSM-5.

### Comparison of cases (all PTSD) and controls (Prolific controls)

Table 2 shows the differences between the Prolific control group and all PTSD group. There were minor differences in age and gender. The case group had experienced an average of five traumas per individual, measured using the LEC-5, compared with two per individual in the control group. There were differences in the scores for the RFQ-8 for the two groups. The case group had both a higher RFQ-U and RFQ-C score than the controls, indicating greater self-reported difficulties with mentalising. There were also differences in the scores on the Modified-STOMP task, with the cases having a higher score on the proportion of ‘Insight’ words (e.g. know) in the total text compared with the controls. There was little difference in the scores for the other social cognition measures.

**Table 2:** Comparison of the all PTSD group and Prolific control group

Characteristics	All PTSD	Prolific controls	
*n*	98	99	

	*n* (%)	*n* (%)	*p*-value^a^
Male gender	24 (24.5)	31 (31.3)	0.286
Autism diagnosis	1 (1.0)	4 (4.0)	0.178

	Median	Median	*p*-value^b^
Age, years	34	33	0.882
STW	50	50	0.941
LEC-5 (experienced)	5	2	<0.001
PCL-5	55	10	<0.001
SST	15	16	0.230
Oddity	29	30	0.517
RMET	27	26	0.889
Modified-STOMP	1.72	1.23	0.079
RFQ-U	1.17	0.33	<0.001
RFQ-C	1	0.33	<0.001

PTSD, post-traumatic stress disorder; STW, Spot-the-Word test; LEC-5, Life Events Checklist for DSM-5; PCL-5, PTSD Checklist for DSM-5; SST, Social Shapes Test; Oddity, Emotion odd-one-out ‘Oddity’ task; RMET, Reading the Mind in the Eyes Task; STOMP, Spontaneous Theory of Mind Task; RFQ-U, Eight-Item Reflective Functioning Questionnaire – hypomentalisation; RFQ-C, Eight-Item Reflective Functioning Questionnaire - hypermentalisation.

a. Chi-squared test.

b. Mann–Whitney *U*-test.

Appendix Table 1 compares the same variables in the Prolific Control, Clinical PTSD and Prolific PTSD groups. This shows that the Clinical PTSD group had the highest PCL-5 score (median: 61 out of a maximum of 80) and thus the most severe PTSD, the prolific PTSD group had a medium score (median: 42), and the Prolific control group had a low score (median: 10).

### Intercorrelation between the social cognition measures

The RFQ-U and the RFQ-C had a very strong positive correlation (rho = 0.93). The SST had a moderate positive correlation with the face emotion Oddity task (rho = 0.33), and a similar positive correlation with the RMET (rho = 0.28). The face emotion Oddity task had a modest positive correlation with the RMET (rho = 0.28) (see Appendix Table 2).

### Logistic regression analyses: social cognition and lifetime exposure to trauma

A higher score on the hypomentalisation questionnaire RFQ-U showed an association with higher odds of trauma (adjusted odds ratio 1.80, 95% CI 1.03–3.14, *p* = 0.040) (see Table 3). There was little evidence of association with trauma for the other social cognition measures.

**Table 3:** Odds ratios, 95% confidence intervals and *p*-values describing the relationship between social cognition and trauma

Measure	Crude			Adjusted^a^		
	Odds ratio	95% CI	*p*-value	Odds ratio	95% CI	*p*-value
SST	1.16	0.75–1.79	0.514	1.25	0.77–2.01	0.366
Oddity	1.33	0.88–1.99	0.172	1.37	0.89–2.11	0.155
RMET	1.48	0.99–2.22	0.054	1.48	0.97–2.27	0.068
Modified-STOMP	1.15	0.69–1.89	0.595	1.10	0.66–1.86	0.711
RFQ-U	1.69	0.98–2.92	0.059	1.80	1.03–3.14	0.040

SST, Social Shapes Test; Oddity, Emotion odd-one-out ‘Oddity’ task; RMET, Reading the Mind in the Eyes Task; STOMP, Spontaneous Theory of Mind Task; RFQ-U, Eight-Item Reflective Functioning Questionnaire - hypomentalisation.

a. Adjusted for gender, age, verbal IQ (Spot-the-Word score) and autism diagnosis.

### Logistic regression analyses: social cognition and PTSD symptoms

Both crude and adjusted results showed a strong association between the self-report hypomentalisation measure RFQ-U (adjusted odds ratio 4.29, 95% CI 2.76–6.66, *p* < 0.001) and PTSD symptoms (see Table 4). A greater proportion of ‘Insight’ words on the Modified-STOMP task was also associated with PTSD symptoms (adjusted odds ratio 1.65, 95% CI 1.17–2.33, *p* = 0.004).

**Table 4:** Odds ratios, 95% confidence intervals and *p*-values describing the relationship between social cognition and PTSD symptoms

Measure	Crude			Adjusted^a^		
	Odds ratio	95% CI	*p*-value	Odds ratio	95% CI	*p*-value
SST	0.91	0.69–1.21	0.532	0.89	0.65–1.22	0.469
Oddity	0.90	0.68–1.19	0.466	0.88	0.65–1.18	0.398
RMET	1.00	0.75–1.32	0.976	0.99	0.73–1.33	0.934
Modified-STOMP	1.61	1.16–2.25	0.005	1.65	1.17–2.33	0.004
RFQ-U	4.15	2.71–6.34	<0.001	4.29	2.76–6.66	<0.001

PTSD, post-traumatic stress disorder; SST, Social Shapes Test; Oddity, Emotion odd-one-out ‘Oddity’ task; RMET, Reading the Mind in the Eyes Task; STOMP, Spontaneous Theory of Mind Task; RFQ-U, Eight-Item Reflective Functioning Questionnaire - hypomentalisation.

a. Adjusted for gender, age, verbal IQ (Spot-the-Word score) and autism diagnosis.

### Sensitivity analysis: social cognition and PTSD symptoms restricted to the Prolific sample

The strong association between the self-report measure RFQ-U and PTSD symptoms remained when analyses were restricted to the Prolific sample (adjusted odds ratio 4.03, 95% CI 2.32–7.04, *p* < 0.001). The association between the Modified-STOMP and PTSD symptoms, however, was reduced to non-significant (adjusted odds ratio 1.52, 95% CI 0.98–2.38, *p* = 0.064) (see Appendix Table 3).

## Discussion

### Principal study findings

This study examined whether different social cognitive indices were associated with trauma and PTSD symptoms. Data were collected at a single time point, from a clinical and non-clinical sample.

We found that our case group reported greater difficulties in mentalising on the RFQ-8. In the logistic regression analyses, there was weak evidence of an association between self-report of hypomentalisation measured using the RFQ-U and experience of a trauma. We found evidence that hypomentalisation measured using the RFQ-8 was associated with significantly increased odds of PTSD symptoms.

We also found an association between greater use of mentalising language on the naturalistic Modified-STOMP task of spontaneous social cognition and PTSD symptoms. This finding, however, did not persist in a sensitivity analysis restricted to the Prolific sample only.

We found no evidence of any significant association between the three performance-based measures of facial emotion processing and mental state attribution (SST, Oddity, RMET) and history of trauma or PTSD symptoms.

### Comparison with previous studies

Previous reviews (including quantitative meta-analyses) have found that social cognition, particularly mentalisation, is impaired in people with PTSD compared with controls.^10–12^ We found that mentalisation differed between the cases and controls using the RFQ-8 and the Modified-STOMP, but not with more constrained performance-based measures of social cognition focused on emotion recognition and attribution.

We found limited intercorrelations between most of the social cognition measures. Interrelations between social cognition measures have typically been found to be modest in previous research,^38^ consistent with the idea that social cognition reflects a range of distinct, domain-specific abilities. We found strong correlation between the RFQ-U and the RFQ-C measures. The RFQ-U and RFQ-C are mainly derived from coding of the same set of questions. Previous work has found that a general mentalising difficulty could result in both hyper- and hypomentalising.^39^ However, confirmatory factor analysis has also shown that the RFQ-8 is a unidimensional measure that most accurately captures hypomentalising.^40^

We found an association between greater use of ‘Insight’ words in the written descriptions of a social movie scene from the Modified-STOMP task and increased odds of PTSD, potentially reflecting hypermentalising. Perhaps consistent with this, previous work found that a greater proportion of ‘Insight’ words in a written trauma account at baseline predicted less trauma symptom reduction over time in a population exposed to a natural disaster.^41^

A recent meta-analytic study has observed an association between mentalising deficits and psychopathology more broadly.^42^ It is possible our findings reflect other psychopathology more common in a PTSD sample rather than PTSD *per se*. This study also found that explicit mentalising, such as self-report rather than task-based measures, had a greater correlation with psychopathology, as we observed.^42^

### Implications and areas for future research

The social–cognitive hypothesis of PTSD theorises that difficulties with social cognition increase the likelihood of PTSD developing following a traumatic life event.^3^ This study has shown an association between mentalising difficulties measured via the RFQ-8 and PTSD. We also found that greater proportions of ‘Insight’ words (reflecting mental state terms like ‘think’ and ‘feel’) used to describe a movie scene (Modified-STOMP) was associated with PTSD on the main analysis, but not the sensitivity analysis restricted to the Prolific sample. This finding initially appears to be the opposite of what we had hypothesised: that lower proportions of ‘Insight’ words would be associated with PTSD. However, use of mental state terms may not necessarily reflect mentalising accuracy, and the finding could reflect hypermentalising: that is, there may have been greater use of mentalising language, but this could have been inaccurate or excessive, given that previous work suggests that increased use of ‘Insight’ words indicate attempts to make sense of events,^43^ which may be more difficult or less automatic in individuals with lower social cognitive ability. The inconsistency between the findings of the main and sensitivity analyses could be attributable to reduced power as a result of either a smaller sample size in the sensitivity analysis, a type 1 error or because the difference in social cognitive ability between the clinical cases and Prolific controls is greater than that between the Prolific cases and Prolific controls.

Another aspect to consider is the difference between reflective functioning/mentalising and theory of mind. These are often conflated concepts in social cognition, but mentalising (as measured using the RFQ-8) looks at awareness of subjective states and mental processes in oneself and others (a meta-social–cognitive ability), whereas theory of mind is the awareness of the experiences of others’ through perspective taking (a social–cognitive ability).^44^ In borderline personality disorder, another psychiatric condition that often includes a significant traumatic component, reflective functioning is impaired to a much greater degree than theory of mind.^44^ There is only weak correlation between self-report and measured social cognitive abilities.^8^

We did not find an association between performance-based measures of social cognition (which covered primarily other aspects of social cognition such as face emotion recognition and emotion attribution to animated shapes) and PTSD. These findings support the hypothesis that specific difficulties in mentalising (reflective functioning), rather than broad social cognition impairments, are associated with PTSD. However, because of the cross-sectional nature of the study, it is hard to determine the direction of causality. Further work is needed in a well-powered longitudinal cohort study to determine if mentalising difficulties measured before trauma exposure are associated with increased risk of developing PTSD. Additional research could examine why mentalisation difficulties are associated with increased likelihood of trauma exposure and PTSD, the latter ideally measured by a clinical interview. If the association between mentalising tendencies and PTSD is found to be robust, this could allow early identification of vulnerable individuals in populations with a heightened risk of exposure to trauma, such as the military or emergency service workers. This could be assessed efficiently in clinical settings with the addition of the RFQ-8 tool.

### Strengths and limitations

A key strength of this study design is the breadth and variety of social cognition measures used, including both self-report questionnaires and constrained and open-ended performance-based tasks. We used a larger sample size than many other studies in the field, which will have increased the power and precision of the findings. Logistic regression allowed us to adjust for several confounder variables.

However, the study must be viewed in the context of several limitations. First, as this is a case–control study, it is problematic to infer causal relationships as the temporality of any relationships present cannot be determined from data collected at a single time point. Therefore, even though our results are consistent with mentalisation difficulties resulting in greater likelihood of PTSD, it is also plausible that trauma exposure and PTSD result in greater difficulties mentalising. Other researchers have suggested that trauma exposure can result in impaired mentalising,^45^ and that this relationship is complex and multifaceted.^46^ Previous qualitative research we have conducted has also hinted at a bidirectional relationship between social cognition and PTSD.^15^

In addition, there is a difference between ability and propensity in social cognitive research.^7^ Most of the tasks used in this study would measure mentalizing ability, or the accuracy of mentalising, rather than an individual’s likelihood to mentalise (propensity). Performance on the measures used can be situation-dependent and laboratory tasks may lack ecological validity.^38^ There is also only weak correlation between self-report and task-based measures of social cognition.^8^

Our cases were selected both from NHS services and from the Prolific sample screened using the PCL-5, and therefore are somewhat heterogenous. We are less confident that the Prolific cases would meet the threshold for a clinical diagnosis of PTSD. More detailed information on trauma histories and chronicity of symptoms for the clinical and Prolific participants would have added to the study analyses. However, as data were collected remotely during the COVID-19 pandemic, we limited data collection on traumatic events and disability to minimise distress. We completed sensitivity analyses restricted to the Prolific sample to manage the heterogeneity.

There are also issues with the data collection techniques. Controlled laboratory conditions were not used for data collection for most participants because of the COVID-19 pandemic restricting face-to-face research. Most study participants were recruited via the online Prolific platform. We chose to recruit from this platform as it has a good reputation and study participants are more honest and naïve then other recruitment platforms.^13,47^ However, social science studies repeated online during the pandemic were found to have a reduced effect size compared with pre-pandemic findings.^48^ Participants completing the study online may have had divided attention or been doing the bare minimum to complete the study and obtain the financial reward (satisficing).^13^ We attempted to manage this by embedding three attention checks into the Prolific tasks and questionnaires. All participants passed at least two of these. However, participants may have been aware of these and looked out for them. Careless responding would likely result in an underestimate of the true effect size.

We had planned to use both subscales of the RFQ-8 to examine the association between hypomentalisation (RFQ-U) and hypermentalisation (RFQ-C) with the outcome variables. However, because of high collinearity between the subscales (rho = 0.93), we decided to only include the RFQ-U scale. Recent confirmatory factor analysis has demonstrated the RFQ-8 is unidimensional and best captures hypomentalising, so we have used it *post hoc* in this context.^40^

The use of multiple measures of social cognition is both a strength and weakness in our study. Looking at multiple measures means we are more likely to have found a type 1 error. The RFQ-8 is also a self-report measure and some of the association seen between could be a result of capturing general psychological distress and symptom severity rather than mentalising difficulties specifically. However, our main association between the RFQ-8 and PTSD has a moderately large effect size and a small *p*-value in both our main and sensitivity analyses, providing evidence of an association (*p* < 0.001).

This study found higher rates of trauma exposure and PTSD prevalence than in the general population. Eighty-five per cent of our Prolific participants reported a trauma exposure compared with a 70% worldwide trauma prevalence.^49^ Thirty per cent of participants screened positive for PTSD in the Prolific sample, higher than the lifetime prevalence of 7%.^50^ It could be that Prolific participants with trauma exposure or PTSD were particularly interested in completing this study, or that the PCL-5, which is a screening tool, may have detected generic psychiatric distress as well as PTSD (i.e. low specificity). This would be consistent with a recent meta-analytic finding that mentalisation difficulties are correlated with psychopathology more broadly.^42^ We did not examine type or number of traumas in detail in our analyses, which may have affected the results.

We only adjusted for four variables (age, gender, verbal IQ, autism status) as confounders in our analyses. Other unmeasured confounders, such as ethnicity and socioeconomic status, could also have influenced the results.

In conclusion, this study used data collected from a clinical and an online sample to examine the relationship between social cognition and PTSD symptoms. We found a significant association between a self-report measure of mentalisation difficulties and PTSD symptoms. We also found some association between an unconstrained movie-based task of spontaneous mentalisation and PTSD symptoms, but this finding was not repeated in a sensitivity analysis restricted to the non-clinical sample. We did not find an association between more constrained performance-based measures of social cognition and the outcome measures. Our findings, despite their limitations, inform social cognitive theories of PTSD.

## Supporting information

10.1192/bjo.2026.12006.sm001 Wiseman et al. supplementary material Wiseman et al. supplementary material

## Data Availability

The anonymised data are available from Dr Sarah Sullivan at The University of Bristol Research Data Storage Facility via the corresponding author.

## Supplementary material

The supplementary material is available online at https://doi.org/10.1192/bjo.2026.12006
