# Supplementary material for: A case–control study examining the relationship between social cognition and post-traumatic stress disorder
Source: BJPsych Open. 2026 Jun 4;12(4):e154. doi: 10.1192/bjo.2026.12006 (PMC13237117; doi:10.1192/bjo.2026.12006)
Supplement: Wiseman et al. supplementary material [file S2056472426120067sup001.docx]

**Appendices**

Appendix Table 1: Comparison of the Clinical-PTSD, Prolific-PTSD and Prolific-Control groups

| **Characteristics** | **Clinical-PTSD** | **Prolific-PTSD** | **Prolific-Controls** |  |
| --- | --- | --- | --- | --- |
| **n** | 56 | 42 | 99 |  |
|  | **N (%)** | **N (%)** | **N (%)** | **P value^a^** |
| **Male sex** | 12 (21.42%) | 12 (28.57%) | 31 (31.31%) | 0.417 |
| **Autism diagnosis** | 0 (0%) | 1 (2.38%) | 4 (4.04%) | 0.306 |
|  | **Median** | **Median** | **Median** | **P value^b^** |
| **Age** | 37.50 | 26 | 33 | 0.042 |
| **STW** | 50 | 50 | 50 | 0.279 |
| **LEC (experienced)** | 6 | 4 | 2 | <0.001 |
| **PCL-5** | 61 | 42 | 10 | <0.001 |
| **SST** | 15 | 16.50 | 16 | 0.118 |
| **Oddity** | 27 | 28 | 30 | 0.285 |
| **RMET** | 28 | 30 | 26 | 0.523 |
| **Modified-STOMP** | 2.04 | 1.33 | 1.23 | 0.009 |
| **RFQ-U** | 1.50 | 1.17 | 0.33 | <0.001 |
| **RFQ-C** | 1.08 | 0.92 | 0.33 | <0.001 |

^a^**Chi-squared test; ^b^ Kruskal Wallis test;**

Appendix Table 2: Correlation between the social cognition measures

| **MEASURE** | **SST** | **Oddity** | **RMET** | **STOMP** | **RFQ-U** | **RFQ-C** |
| --- | --- | --- | --- | --- | --- | --- |
| **Oddity** | 0.33  p<0.001 | 1.00 | - | - | - | - |
| **RMET** | 0.28  p<0.001 | 0.28  p<0.001 | 1.00 | - | - | - |
| **Modified-STOMP** | -0.14  p=0.054 | -0.18  p=0.014 | -0.01  p=0.907 | 1.00 | - | - |
| **RFQ-U** | -0.08  p=0.243 | -0.03  p=0.972 | -0.09  p=0.189 | 0.13  p=0.068 | 1.00 | - |
| **RFQ-C** | -0.11  p=0.116 | -0.03  p=0.649 | -0.16  p=0.021 | 0.13  p=0.061 | 0.93  p<0.001 | 1.00 |

Appendix Table 3: Odds ratios, 95% confidence intervals and p values describing the relationship between social cognition and PTSD symptoms restricted to the Prolific sample. Crude and adjusted results shown.

|  | **Crude** | | | **Adjusted*** | | |
| --- | --- | --- | --- | --- | --- | --- |
| **Measure** | **OR** | **95% CI** | **P value** | **OR** | **95% CI** | **P value** |
| **SST** | 1.05 | 0.74, 1.48 | 0.803 | 0.86 | 0.59, 1.27 | 0.456 |
| **Oddity** | 1.10 | 0.74, 1.66 | 0.636 | 1.00 | 0.65, 1.52 | 0.990 |
| **RMET** | 1.18 | 0.79, 1.76 | 0.411 | 1.14 | 0.76, 1.70 | 0.524 |
| **Modified-STOMP** | 1.40 | 0.92, 2.13 | 0.121 | 1.52 | 0.98, 2.38 | 0.064 |
| **RFQ-U** | 3.92 | 2.33, 6.77 | <0.001 | 4.03 | 2.32, 7.04 | <0.001 |

*Adjusted for sex, age, verbal IQ (STW score) and autism diagnosis
